# Supplementary material for: Mechanisms of the RNA helicases DDX42 and DDX46 in human U2 snRNP assembly
Source: Nat Commun. 2023 Feb 17;14:897. doi: 10.1038/s41467-023-36489-x (PMC9935549; doi:10.1038/s41467-023-36489-x)
Supplement: Supplementary file 1 — Supplementary Information [file 41467_2023_36489_MOESM1_ESM.pdf]

Supplementary Information

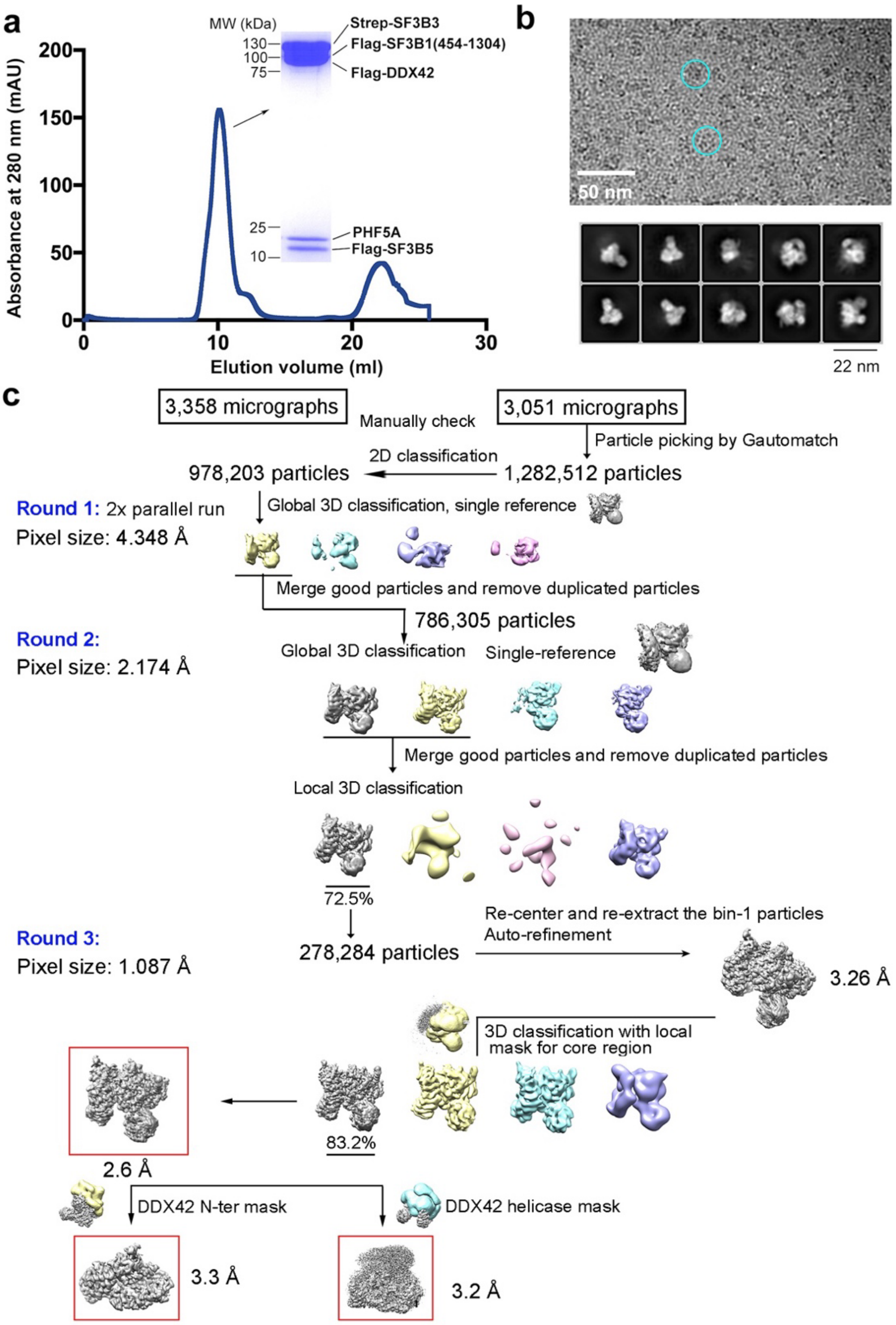

Supplementary Figure 1. Purification and characterization of the DDX42-SF3b complex. **a**, The purified DDX42-SF3b complex is eluted from gel filtration as a well-behaved peak. The peak fraction was visualized on an SDS-PAGE gel and stained by Coomassie blue. Experiments were repeated three times with similar results. **b**, A representative cryo-EM micrograph (upper panel) and representative 2D class averages (lower panel) of the human DDX42-SF3b complex. **c**, A flow chart diagram of cryo-EM data processing for the DDX42-SF3b core complex. Please refer to Methods for details.

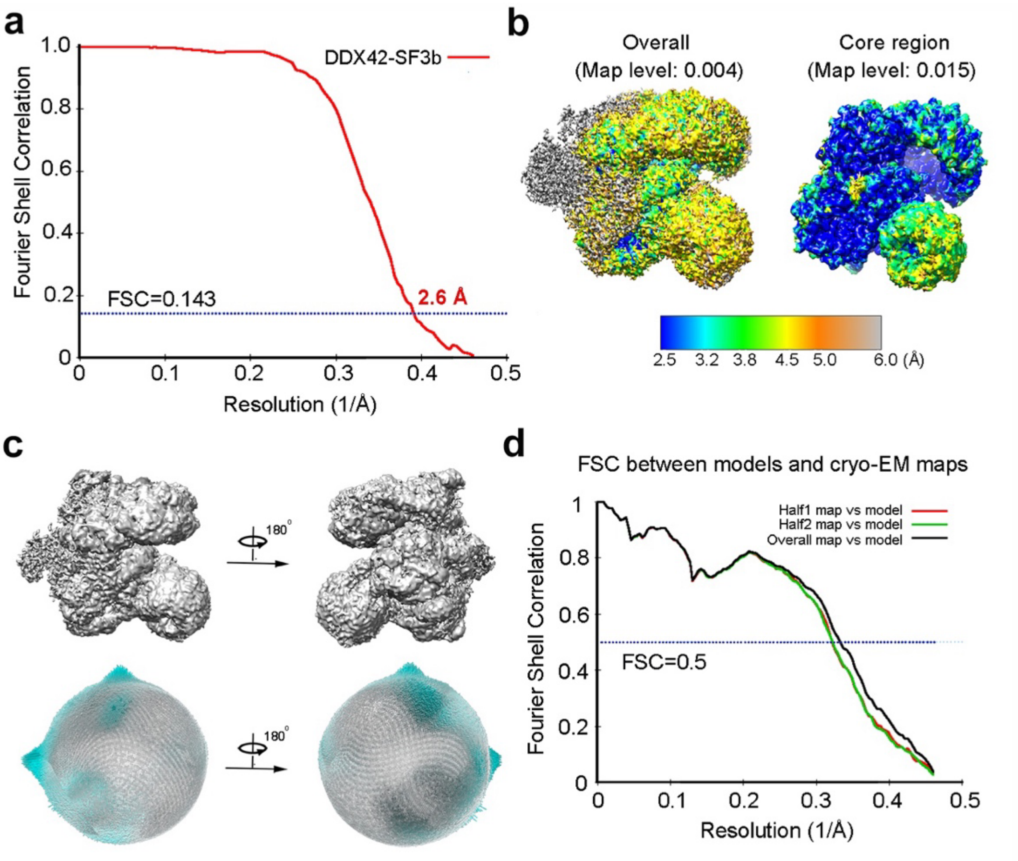

Supplementary Figure 2. The cryo-EM reconstruction of the DDX42-SF3b complex at an average resolution of 2.6 Å. **a**, The average resolution of the overall EM map is estimated to be 2.6 Å on the basis of the FSC criterion of 0.143. **b**, An overall view of the EM density maps. The overall EM map (left panel) and the EM map for the core region (right panel) are shown. The local resolutions are color-coded for different regions of the DDX42-SF3b core complex. **c**, Angular distribution of the particles used for the reconstruction. Each cylinder represents one view, and the height of the cylinder is proportional to the number of particles for that view. **d**, The Fourier-shell correlation (FSC) curves for cross-validation between the model and the cryo-EM map for the DDX42-SF3b complex. Shown here are the FSC curves between the final refined atomic model and the reconstruction from all particles (black), between the model refined in the reconstruction from only half of the particles and the reconstruction from that same half (red), and between that same model and the reconstruction from the other half of the particles (green).

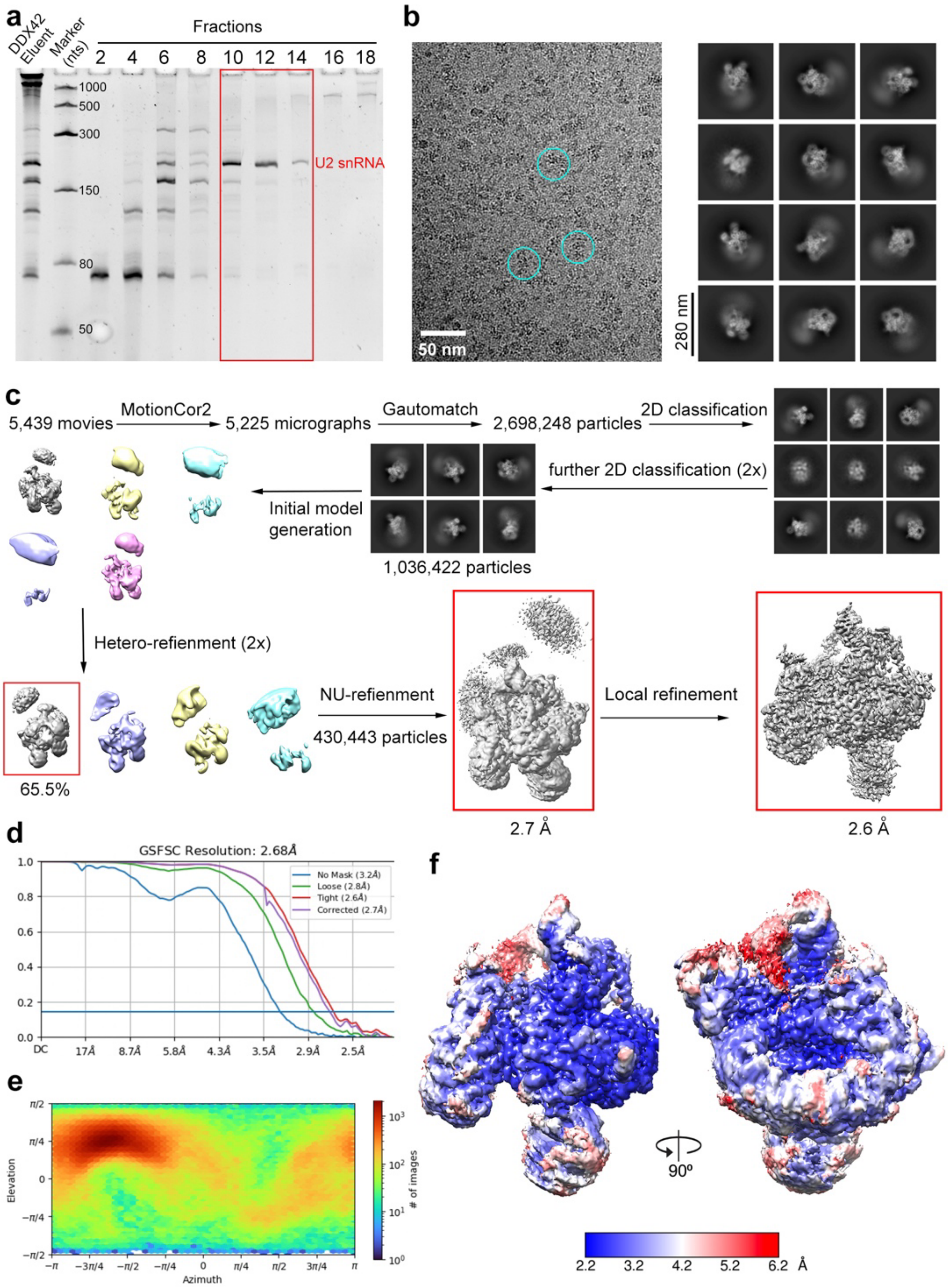

Supplementary Figure 3. Purification and characterization of the DDX42-U2 complex. **a**, The eluent of Flag-DDX42 was applied to glycerol gradient centrifugation. Fractions 10-14, mainly contains U2 snRNA as shown in this denaturing PAGE gel stained by SYBR-Gold, were collected for cryo-EM sample preparation. **b**, A representative cryo-EM micrograph (left panel) and representative 2D class averages (right panel) of the DDX42-U2 complex sample. **c**, A flow chart diagram of cryo-EM data processing for the DDX42-U2 complex. Please refer to Methods for details. **d**, The final reconstruction has an average resolution of 2.7 Å as determined by the FSC value of 0.143. **e**, Angular distribution of the particles in the final round of 3D refinement using CryoSPARC[1]. **f**, Two overall views of the EM density map. The local resolution of the EM density map is color-coded. The analyses in panels **a** and **b** were repeated for three times with similar results.

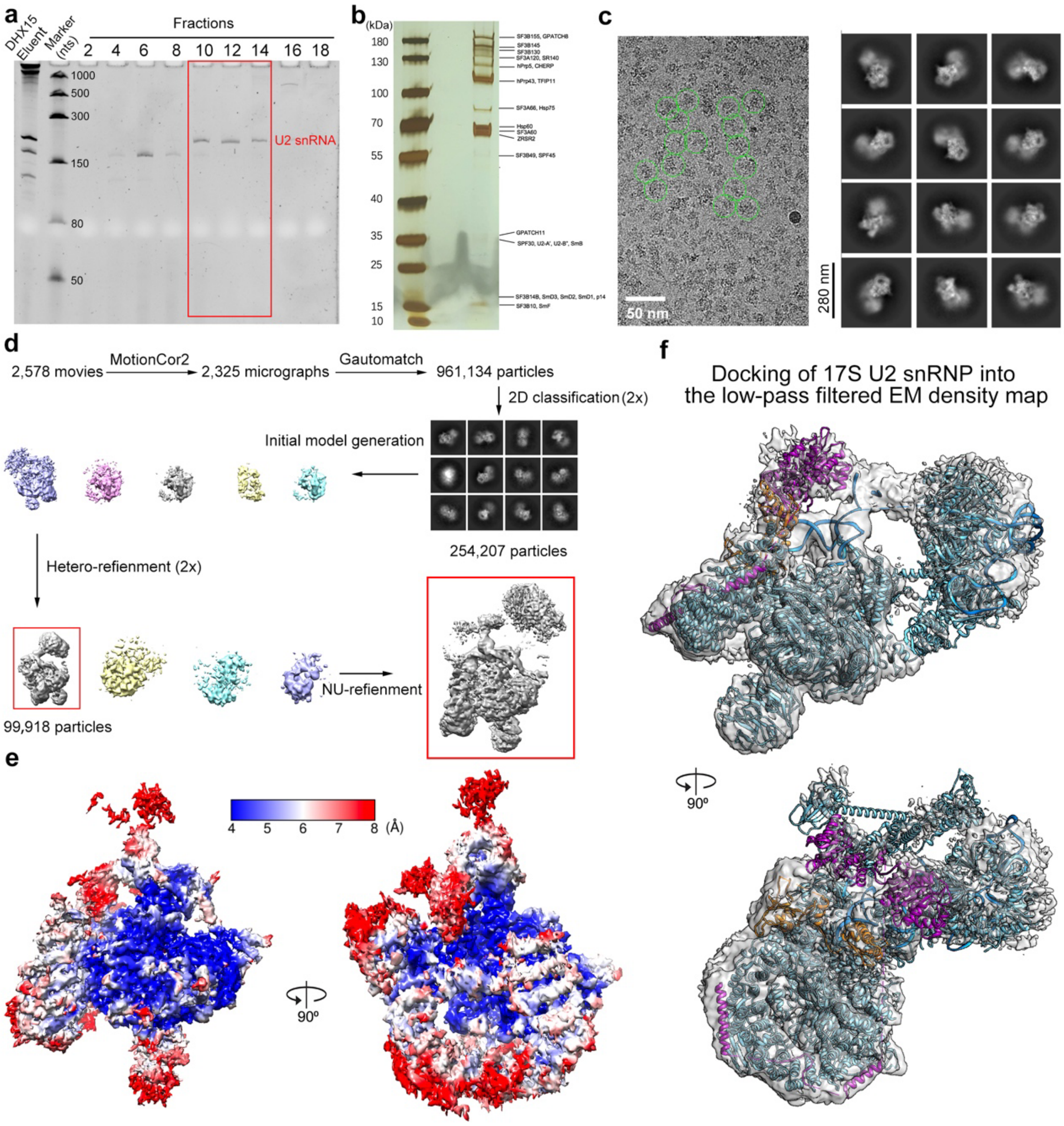

Supplementary Figure 4. Purification and characterization of the DHX15-U2 complex. **a**, The eluent of Flag-DHX15 was applied to glycerol gradient centrifugation. Fractions 10-14, mainly contains U2 snRNA, were collected for cryo-EM sample preparation. **b**, Analysis of the purified sample on a silver-stained SDS-PAGE gel. The protein components were identified by mass spectrometry. DHX15 and serval G-patch proteins, such as TFIP11 and CHERP, were detected in the final sample. **c**, A representative cryo-EM micrograph (left panel) and representative 2D class averages (right panel) of the DHX15-U2 complex. **d**, A flow chart diagram of cryo-EM data processing for the DHX15-U2 complex. **e**, Two overall views of the EM density map. The local resolution of the EM density map is color-coded. **f**, Docking of the atomic model of 17S U2 snRNP[2] into a low-pass filtered EM map of the DHX15-U2 complex. The analyses in panels **a**-**c** were repeated for three times with similar results.

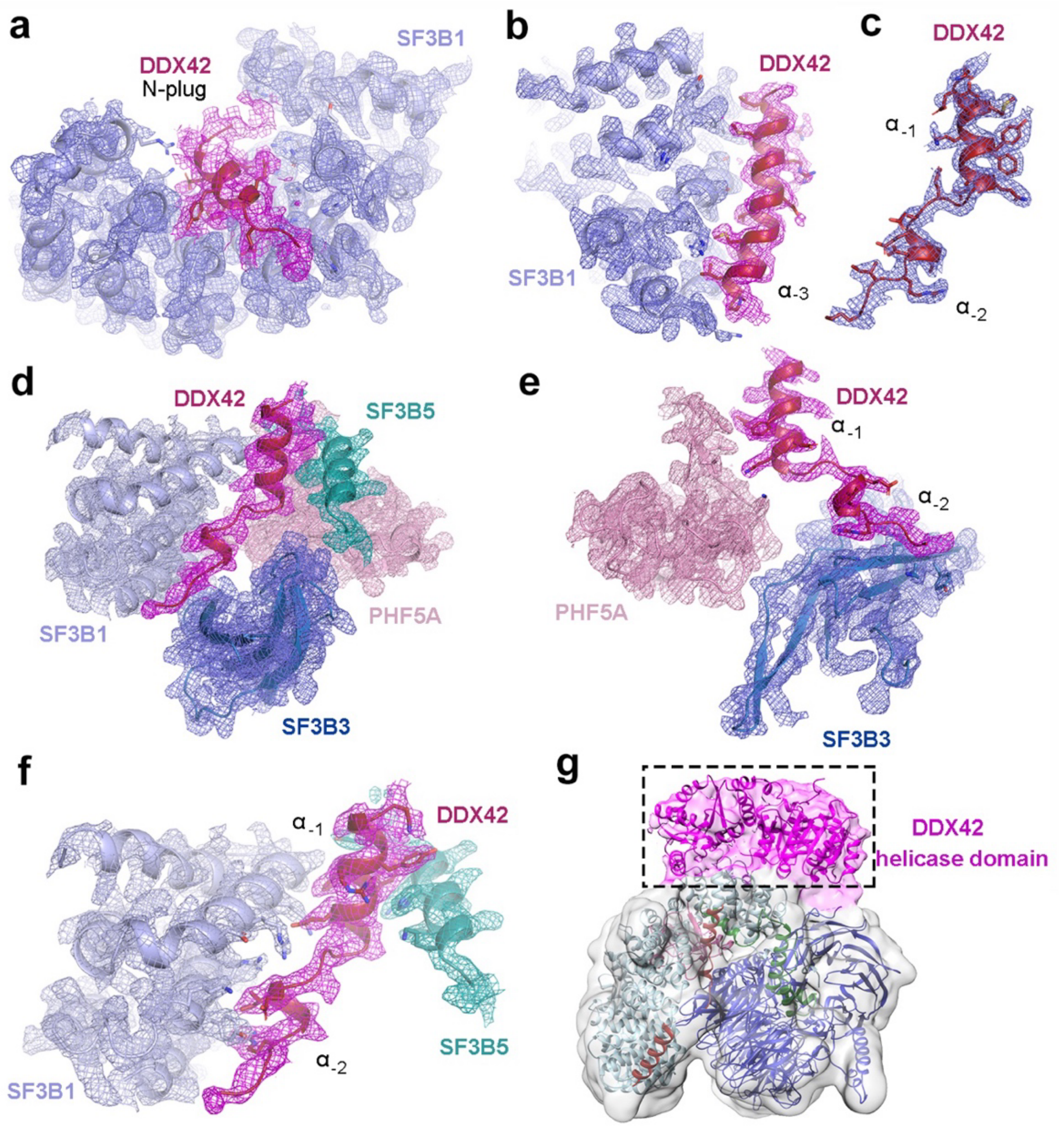

Supplementary Figure 5. Quality of the EM density map for the interfaces between DDX42 and SF3b complex. **a**, The EM density map for the N-plug of DDX42 and its surrounding structural elements of SF3B1. **b**, The EM density map for the helix α_{-3} of DDX42 and its surrounding structural elements of SF3B1. **c**, The EM density map for the two helices α_{-2} and α_{-1} of DDX42. **d**, The EM density map for the two helices α_{-2}/α_{-1} of DDX42 and their surrounding structural elements. **e**, A close-up view on the EM density map of the interface between the helices α_{-2}/α_{-1} of DDX42 and PHF5A/SF3B3. **f**, A close-up view on the EM density map for the helices α_{-2}/α_{-1} of DDX42 and SF3B1/SF3B5. **g**, Docking of the DDX42 helicase domain (colored magenta) into the EM density lobe at the periphery of the DDX42-SF3b core complex.

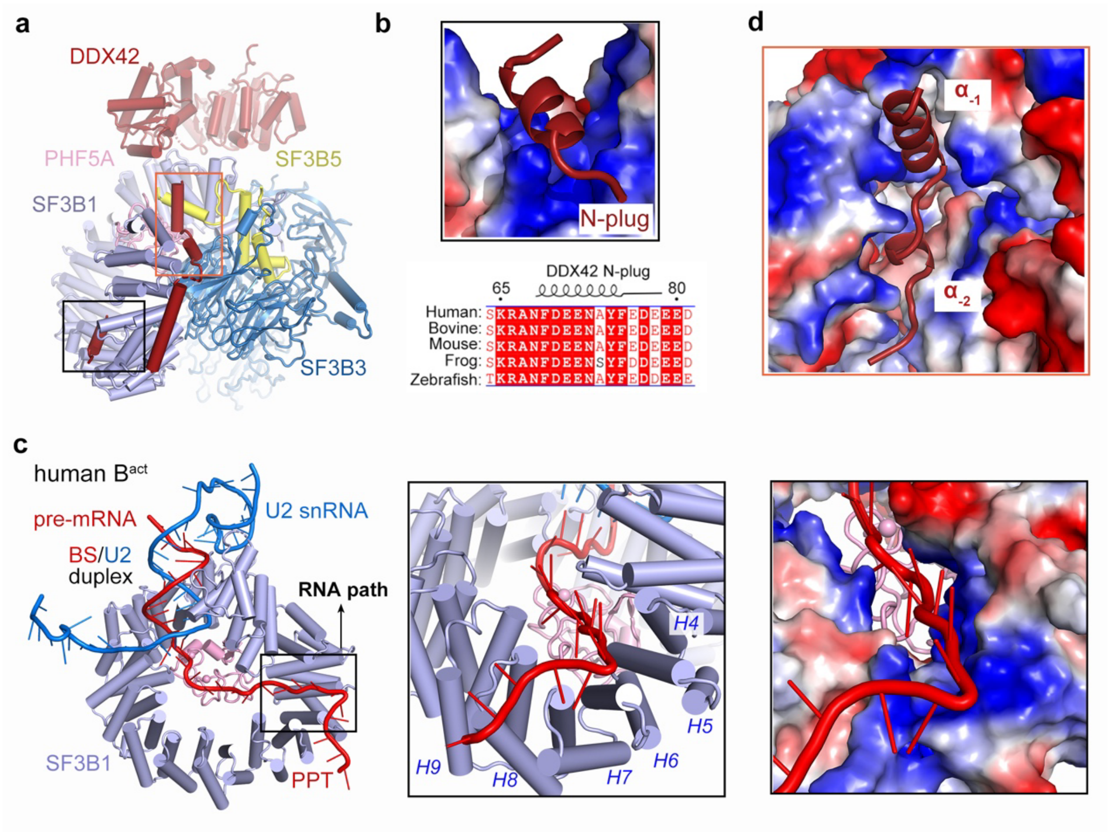

Supplementary Figure 6. DDX42 is anchored on SF3B1 mainly through its N-terminal motifs, with its N-plug occupying the RNA path of SF3B1. **a**, An overall view of the structural organization of DDX42 in the SF3b complex. **b**, The N-plug of DDX42 is accommodated in the positively charged RNA path on SF3B1 (upper panel). The N-plug sequences of DDX42 are highly conserved from zebrafish to humans (lower panel). **c**, Conformation of the RNA elements in U2 snRNP of the human B^{act} complex (PDB code: 5Z56) [3](left panel). The pre-mRNA (red) forms the BS/U2 duplex with U2 snRNA. A close-up view on the pre-mRNA element bound to the RNA path of SF3B1 in the human B^{act} complex (middle panel). The positively charged surface of the RNA path surrounds the negatively charged polypyrimidine tract (PPT) of pre-mRNA in the human B^{act} complex (right panel). **d**, The acidic α_{-1} and α_{-2} helices of DDX42 are bound in a positively charged cleft.

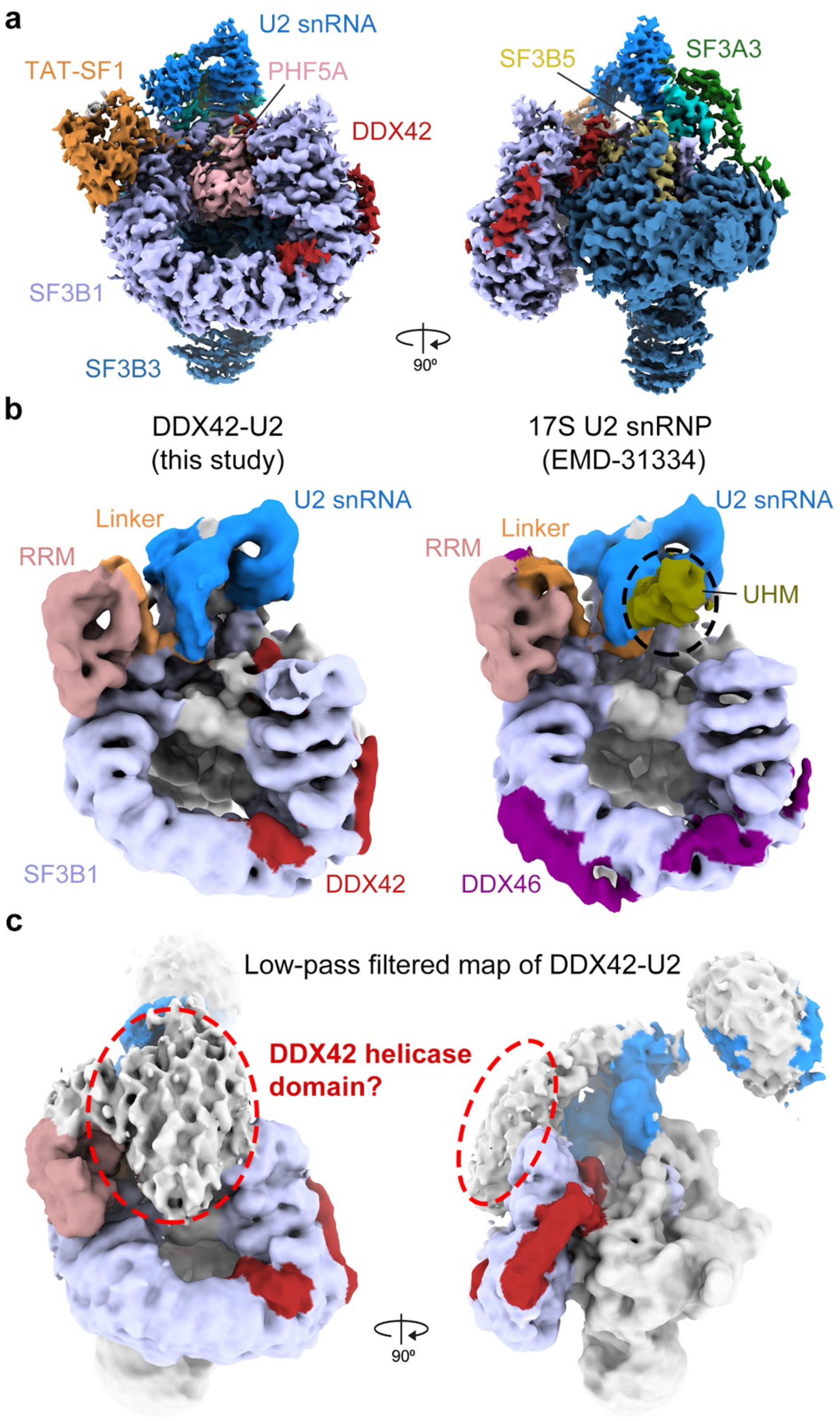

Supplementary Figure 7. Features of the EM density map for the DDX42-U2 complex. **a**, The high-resolution EM map for the core region of human DDX42-U2 complex. The N-terminus, but not the helicase domain of DDX42, was identified in DDX42-U2 complex. **b**, Comparison of the low-pass filtered maps for DDX42-U2 complex (this study) and 17S U2 snRNP (EMD-31334)[2]. Notably, there's no obvious density for the UHM domain of TAT-SF1 in DDX42-U2 complex. **c**, An unassigned EM density lobe (circled with dashed lines) is present in the DDX42-U2 complex. It's unclear whether this lobe belongs to the helicase domain of DDX42.

Supplementary Table 1. Statistics of 3D reconstructions and model refinement

	DDX42-SF3b complex	DDX42-U2 complex
**Data collection**		
EM equipment	FEI Titan Krios	
Voltage (kV)	300	
Detector	K3	
Pixel size (Å)	1.087	
Electron dose (e-/Å^2)	50	
Defocus range (μm)	1.5~2.0	
**Reconstruction**		
Software	RELION 3.0	cryoSPARC
EMDB code	EMD-31330	EMD-34841
Number of particles	234,800	430,443
Symmetry	C1	
Final masked resolution (Å)	2.6	2.7
Map sharpening B-factor (Å^2)	−78.6	-108.5
**Model building**		
Software	Coot 0.8.9	
Refinement	Phenix/Refmac	
PDB code	PDB: 7EVN	PDB: 8HK1
Protein residues	2696	4037
RNA nucleotides	/	148
**Validation**		
R.m.s deviations		
Bonds length (Å)	0.01	0.01
Bonds Angle (°)	0.85	1.72
Ramachandran plot statistics (%)		
Preferred	95.02	91.01
Allowed	4.79	8.38
Outlier	0.19	0.61

Supplementary Table 2. Summary of model building for the DDX42-SF3b complex and DDX42-U2 complex.

	Molecule	Length	Domain/Region	PDB code	Modeling	Resolution (Å)	Chain ID
**DDX42-SF3b complex**	SF3B1	1304	490:1304	5YZA	RD	2.5~3.0	C
	SF3B3	1217	1:1217		RD	2.5~3.0	A
	SF3B5	86	4:81		RD	2.5~3.0	B
	PHF5A	110	6:98		RD	2.5~3.0	D
	DDX42	938	69:167<br>helicase domain	/	DM<br>RD	2.5~3.2<br>5.5~8.0	E
**DDX42-U2 complex**	U2 snRNA	188 nt	12:73/81:184	7EVO	RD	3.5~8.0	H
	SF3B1	1304	490:1304		RD	2.5~3.0	1
	SF3B2	895	458:598	7EVO	RD	2.5~3.0	2
	SF3B3	1217	1:1217	7EVO	RD	2.5~3.0	3
	SF3B4	424	11:181		RD	6.0~10.0	4
	SF3B5	86	16:81		RD	2.5~3.0	5
	PHF5A	110	6:91		RD	2.5~3.0	6
	SF3A1	793	160:282		RD	6.0~10.0	A
	SF3A2	464	104:209		RD	6.0~10.0	B
	SF3A3	501	1:501		RD	6.0~10.0	C
	U2-A’	255	LRR domain		RD	6.0~10.0	F
	U2-B”	225	RRM domain		RD	6.0~10.0	G
	SmB,D1,D2<br>D3,E,F,G	-	Sm fold		RD	6.0~10.0	a-g
	TAT-SF1	755	127:256	/	DM	3.0~4.0	D
	DDX42	938	152:243	/	DM	2.5~3.5	E

Under the column labeled “Modeling”, DM stands for *de novo* modeling; RD stands for rigid docking and manual adjustment.

Supplementary Table 3. Summary of proteins identified in the purified sample of DDX42-U2 complex.

Proteins identified	M.W. (kDa)	Accession No.	Peptides
DDX42-U2 complex proteins			
DDX42	103	Q86XP3	58
SF3B1	146	O75533	58
SF3B3	136	Q15393	52
TAT-SF1	86	O43719	31
SF3B2	100	Q13435	26
SF3A3	59	Q12874	21
SF3A1	89	Q15459	24
SNRPA1	28	P09661	19
SF3A2	49	Q15428	14
SF3B4	44	Q15427	11
SNRPB2	26	P08579	9
SNRPD2	14	P62316	9
SF3B5	10	Q9BWJ5	8
SF3B6	15	Q9Y3B4	7
SNRPB	25	P14678	7
SNRPD3	14	P62318	5
PHF5A	12	Q7RTV0	5
SNRPE	11	P62304	3
SNRPF	10	P62306	3
Ribosomal proteins, chaperons, and other proteins			
EIF3A	166	Q14152	40
HSPA1B	70	A0A0G2JIW1	35
EFTUD2	109	Q15029	29
EIF3C	105	Q99613	24
HSPA8	71	P11142	19
EIF3B	92	P55884	18
HSP90AB1	83	P08238	16
BOP1	84	Q14137	10
LAS1L	83	Q9Y4W2	7
WDR12	50	Q9GZL7	5
MRPS22	41	P82650	4
RPL18	19	H0YHA7	3
RPL22	15	P35268	3

Supplementary Table 4. Summary of key residues in SF3B1 that interact with DDX42 and DDX46.

	SF3B1 residues	DDX42-interacting	DDX46-interacting	Cancer-derived mutation
SF3B1 *H10-H12*	W938		E157	
	N931		R163	
	E902		K164	✓
	Y898		R171	✓
	D894		R163, R166	✓
	E860		R171	
SF3B1 RNA path (*H4-H9*)	R828	E71		
	E783		L200	✓
	D781		K196, W198	✓
	K748	E72	E201	
	K741	Y75, D78	S199, D202, D240	✓
	K700	E79, E80	D203	✓
	Q699	E79	D205	✓
	K666	E77	D203	✓
	H662	E77	D203	✓
	R625	E80	D203	✓
SF3B1 *H3-H5*	K656	D114, E117	D226, D229	
	V651	L116	L228	
	F647	F119	Y231	
	I610	V123	V235	
	R568	D130		
SF3B1 *H1-H2*	R562	E152		
	R558	I151		
	D557	R148		
	R517	D155, E158		
	K513	D155		
	P510	F161		

Residues from DDX42 and DDX46 that interact with SF3B1 are indicated in red and purple, respectively. Residues of SF3B1 that are frequently mutated in cancers are indicated.

Supplementary References

1 DiIorio, M. C. & Kulczyk, A. W. A Robust Single-Particle Cryo-Electron Microscopy (cryo-EM) Processing Workflow with cryoSPARC, RELION, and Scipion. *J Vis Exp*, doi:10.3791/63387 (2022).

2 Zhang, X. Z., X.; Bian, T.; Yang, F.; Li, P.; Lu, Y.; Xing, Z.; Zhang, Q.; Shi, Y. Structural Insights into Branch Site Proofreading by Human Spliceosome. *bioRxiv*, doi:https://doi.org/10.1101/2022.11.07.515429 (2022).

3 Zhang, X. *et al.* Structure of the human activated spliceosome in three conformational states. *Cell Res* **28**, 307-322, doi:10.1038/cr.2018.14 (2018).
